# Supplementary figures and images for: In silico investigation of cytochrome bc1 molecular inhibition mechanism against Trypanosoma cruzi
Source: PLoS Negl Trop Dis. 2023 Jan 23;17(1):e0010545. doi: 10.1371/journal.pntd.0010545 (PMC9894551; doi:10.1371/journal.pntd.0010545)

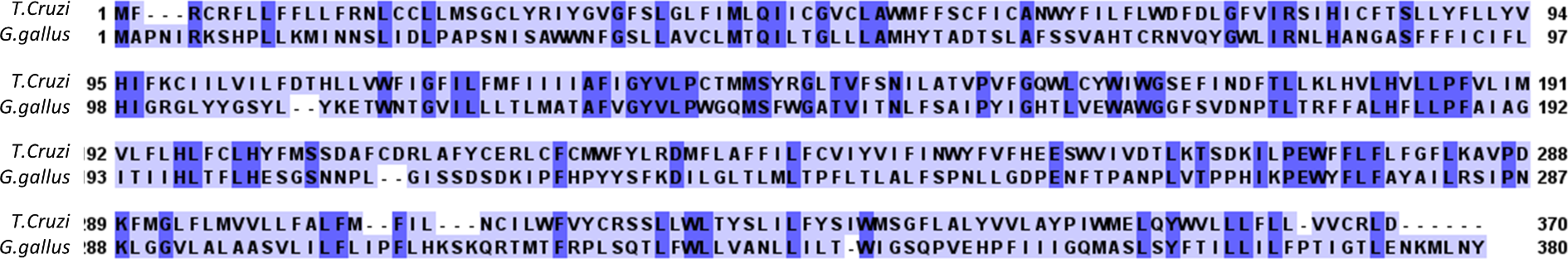

Supplement: S1 Fig — Sequence alignment of cytochrome b of T. cruzi, and G. gallus. (TIFF) [file pntd.0010545.s002.tiff]

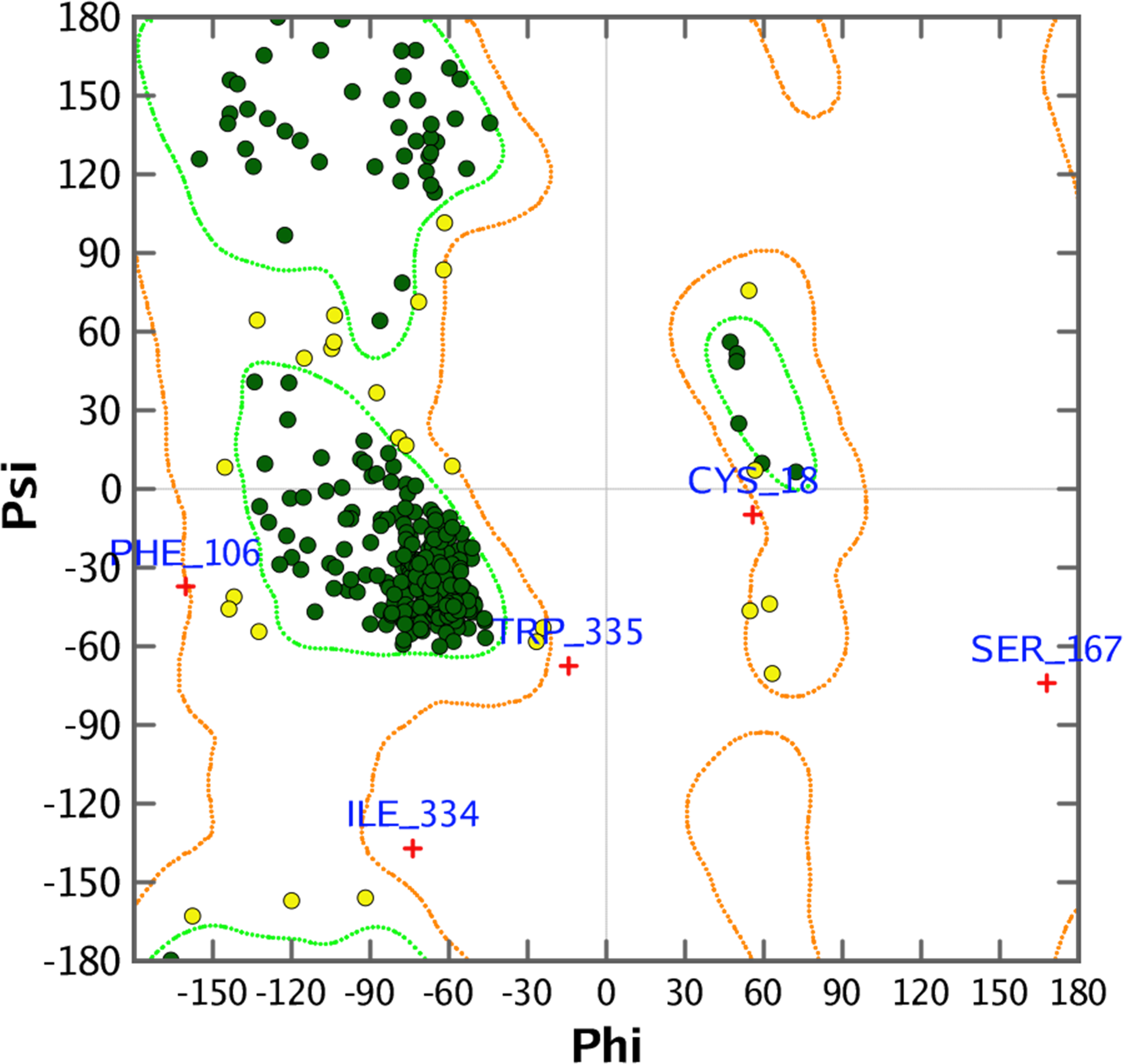

Supplement: S2 Fig — Psi-phi angle in degree of the cytochrome b homology model built. (TIFF) [file pntd.0010545.s003.tiff]

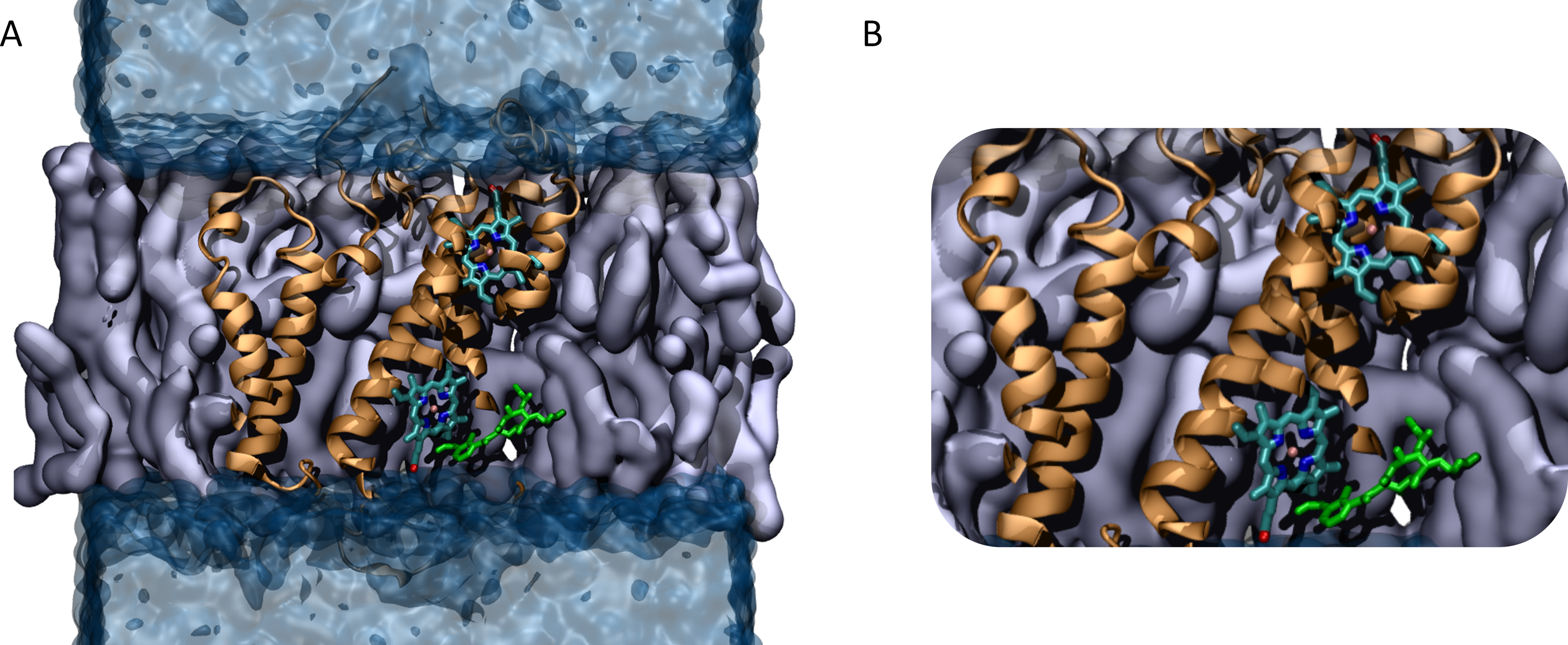

Supplement: S3 Fig — A) Representative complex model built and B) an enlargement of the ligand and heme groups. The cytochrome b is shown in orange, the POPC lipid bilayer in violet, the heme groups in cyan and the ligand in the Qi site in green. (TIFF) [file pntd.0010545.s004.tiff]

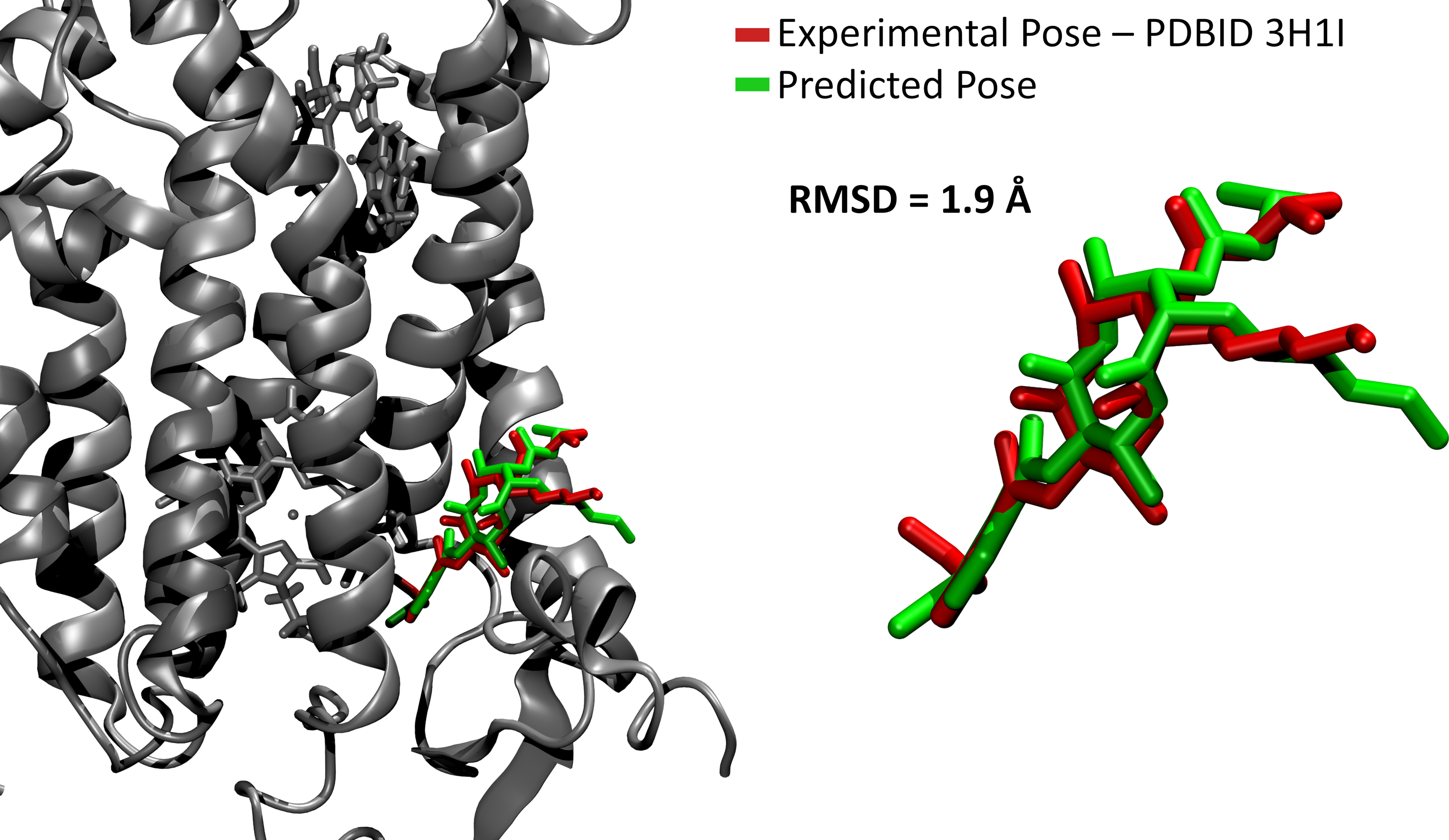

Supplement: S4 Fig — Molecular docking pose validation of the antimycin A in the Qi site. (TIFF) [file pntd.0010545.s005.tiff]
